# Thermal radiation of Er doped dielectric crystals: Probing the range of applicability of the Kirchhoff’s law

**DOI:** 10.1038/s41598-017-01544-3

**Published:** 2017-05-17

**Authors:** Ekembu K. Tanyi, Brandi T. Burton, Evgenii E. Narimanov, M. A. Noginov

**Affiliations:** 1 Center for Materials Research, Norfolk State University, Norfolk, VA 23504 USA; 2 Birck Nanotechnology Center, Department of Electrical and Computer Engineering, Purdue University, West Lafayette, IN 47907 USA

## Abstract

Kirchhoff’s law of thermal radiation, relating emissivity and absorptance is commonly formulated for opaque bodies in thermodynamic equilibrium with the environment. However, in many systems of practical importance, both assumptions are often not satisfied. We revisit the century-old law and examine the limits of its applicability in an example of Er:YAG and Er:YLF dielectric crystals–potential radiation converters for thermophotovoltaic applications. The (80 at.%) Er:YAG crystal is opaque between 1.45 μm and 1.64 μm. In this spectral range, its absorptance α(λ) is spectrally flat and differentiates from unity only by a small amount of reflection. The shape of the emissivity spectrum ɛ(λ) closely matches that of absorptance α(λ), implying that the Kirchhoff’s law can adequately describe thermal radiation of opaque bodies, even if thermodynamic equilibrium is not satisfied. The (20 at.%) Er:YLF crystal had smaller size, lower concentration of Er ions, and it was not opaque. Nevertheless, its spectrum of emissivity had almost the same shape (between 1.45 μm and 1.62 μm) as the absorptance derived from the transmission measurements. Our results are consistent with the conclusion that the Kirchhoff’s law of thermal radiation can be extended (with caution) to not-opaque bodies away from the thermodynamic equilibrium.

## Introduction

### Photovoltaics and thermophotovoltaics

**Figure 1:**
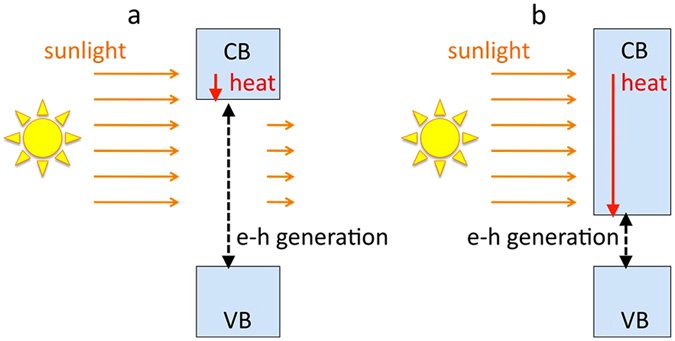
Schematics of generation of electron-hole (**e**–**h**) pairs, showing low efficiency of light absorption in a large-gap semiconductor (**a**) and large heat production in a low-gap semiconductor (**b**). (VB and CB stand for valence band and conduction band, respectively).

In recent years, the development of technologies aimed at harvesting renewable energy, in particular solar energy^1, 2^, has become critically important. The solar energy, most commonly, is absorbed and converted to electricity in a p-n junction semiconductor device known as photovoltaic solar cell^3^. However, photovoltaic cells face an inherent dilemma. Thus, if the bandgap of the semiconductor is large, then a significant portion of the (long-wavelength) sunlight radiation cannot be absorbed, reducing the efficiency of the energy production (Fig. 1a). On the other hand, in a small-bandgap semiconductor, large fraction of the absorbed photons’ energy is converted to heat that lowers the efficiency of the light-electricity conversion as well (Fig. 1b). In the seminal publication^4^, Shockley and Queisser have shown that for the sunlight spectrum approximated by the blackbody radiation at T = 6000 K, the ultimate efficiency of the light-to-electrical energy conversion by a single p-n junction device is equal to only 44%, at the optimal semiconductor bandgap equal to 1.1 eV (very close to that of Si^5^) and the solar cell temperature equal to 0 K. This value is reduced down to ~31% at the bandgap equal to 1.36 eV, if the realistic temperature, impedance matching, geometrical factors, etc. are taken into account^4^.

The Shockley-Queisser limit can be overcome in tandem schemes, in which solar cells with smaller band gaps are placed behind the solar cells with larger band gaps^6–10^. However, technological complexity and high cost prohibit widespread commercial use of such devices.

**Figure 2:**
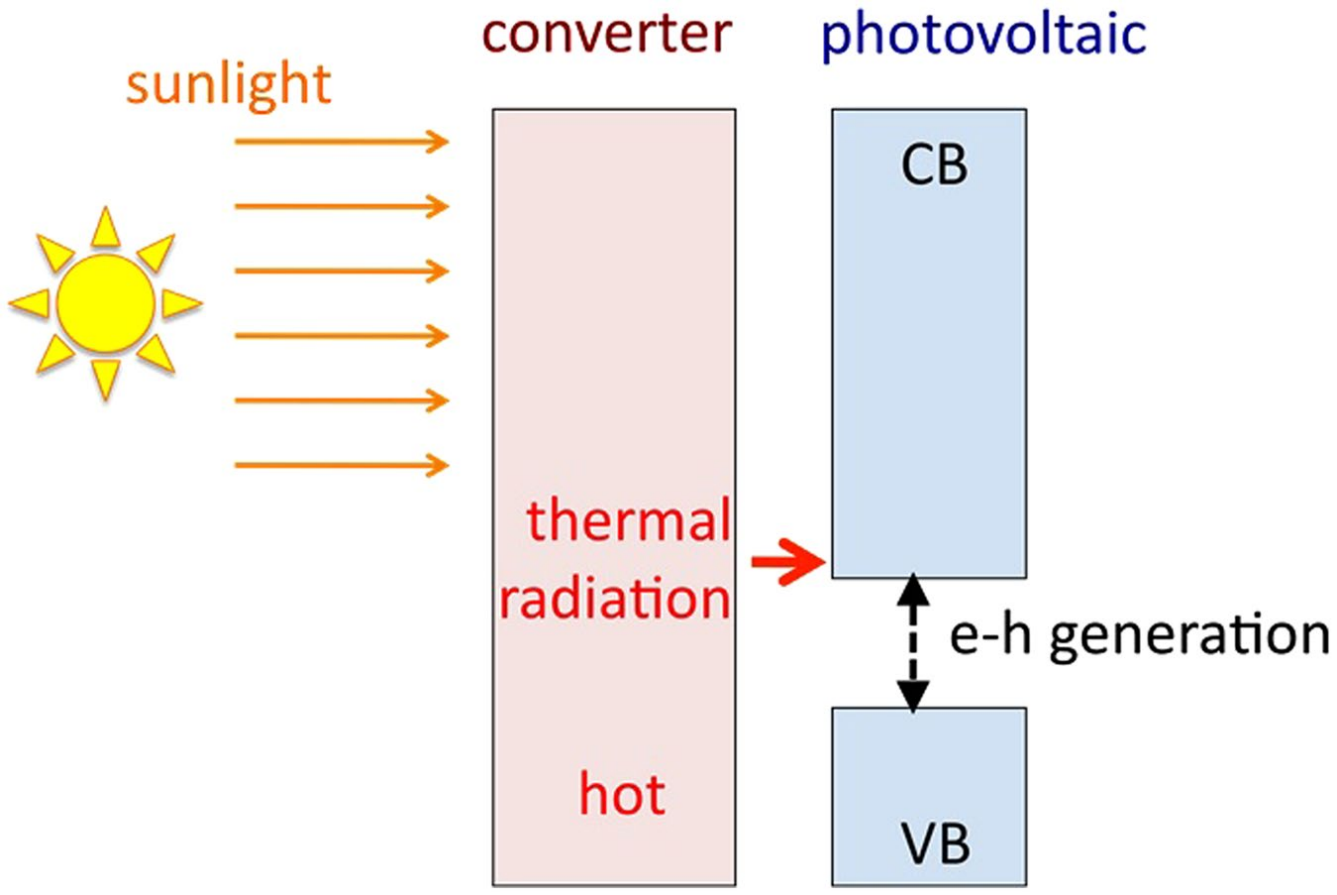
Schematics of a thermophotovoltaic device.

An ideal photovoltaic cell should harvest solar energy in the whole spectrum and utilize most of it to create electron-hole (e–h) pairs. Such functionality can be achieved in a low-gap semiconductor combined with so-called thermophotovoltaic converter^11–18^. The role of the converter is to absorb the solar energy over a broad spectrum, become hot, and reemit the absorbed energy in form of thermal radiation in a narrow spectral band matching the bottom of the semiconductor’s conduction band^19^, Fig. 2. A significant effort has been made to design sub-wavelength patterned surfaces (metasurfaces)^20–24^ or bulk engineered composite materials (metamaterials)^25–33^ able of absorbing solar radiation on one side of the converter and reemitting in a narrow infrared band on the other side.

**Figure 3:**
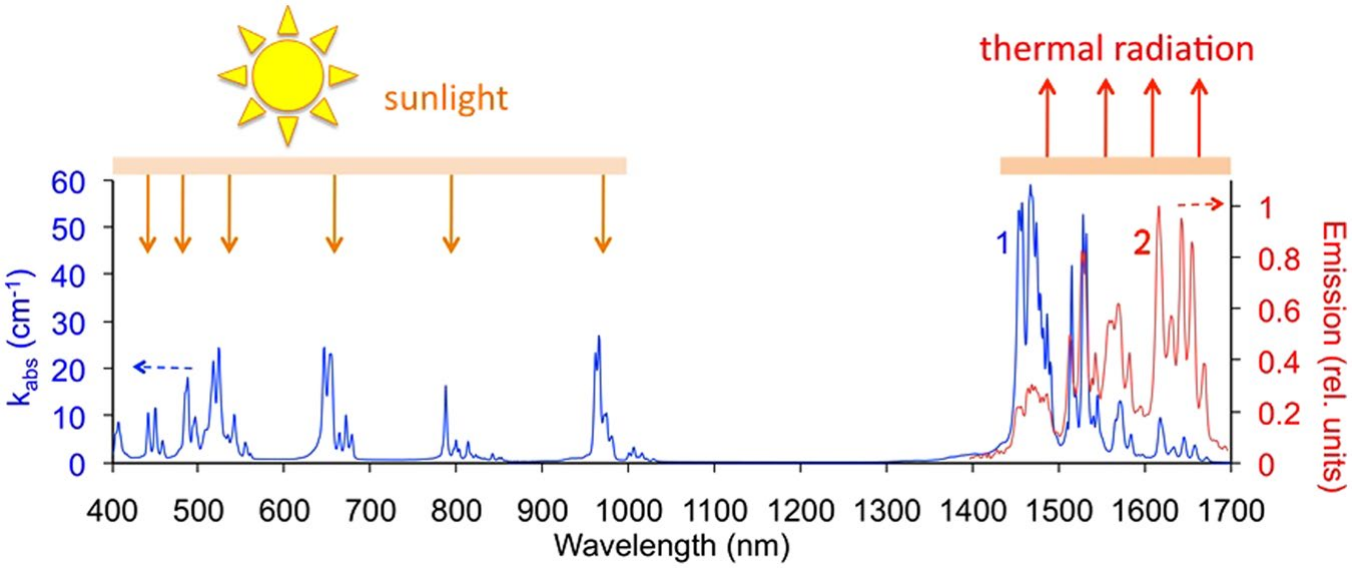
Room temperature absorption (blue trace 1) and emission (red trace 2) spectra of (80 at.%) Er:YAG; optical path *l* = 1.22 mm.

Alternatively, thermal converter of a thermophotovoltaic device can be based on an inorganic crystal doped with rare-earth^34–38^ and transition metal ions^36–39^. Refractory oxide crystals of the garnet family doped with Er^3+^ ions are appealing for thermophotovoltaic applications^34–37^ because they have strong absorption in the visible part of the spectrum, strong emission at ~1.55 μm (Fig. 3), and high melting temperature (1965 °C for Er:Y_3_Al_5_O_12_)^40–42^. Even higher absorption efficiency can be achieved in Er^3+^ doped crystals, such as yttrium scandium gallium garnet (Y_3_Sc_2_Ga_3_O_12_ or YSGG), co-doped by Cr^3+^ ions^41, 43^. Other rare-earth ions of potential importance to thermophotovoltaics have strong absorption and emission bands at ~1.8 μm (Tm^3+^), ~2.0 μm (Ho^3+^) and ~3.4 μm (Dy^3+^)^40, 41, 44^. The focus of this paper is on the crystals doped with Er^3+^ ions.

### Rare-earth based thermophotovoltaic converters: Kirchhoff’s law of emission and absorption

Of particular importance are the absorption and emission spectra of a thermal converter. These functions are related to each other by the Kirchhoff’s law^45^ stating that for *opaque* bodies in *thermodynamic equilibrium* ^46–48^, the radiant emissivity *ε* (defined as the ratio of emitted radiant power *P* to that from an ideal blackbody *P*_*BB*_ at the same temperature) is equal to radiant absorptance *α* (defined as the ratio of absorbed radiant power to incident radiant power), *ε* = *α*. The latter quantity is equal to *α* = 1 − *ρ* − *τ*, where *ρ* is the radiant reflectance (the ratio of absorbed radiant power to incident radiant power) and *τ* is the radiant transmittance (the ratio of transmitted radiant power to incident radiant power); in opaque bodies, *τ* = 0. This law can be formulated for each particular temperature *T*, frequency *ω*, polarization *φ*, and polar coordinates (*θ*, *ϕ*) defining the angle of incidence^49^:

1a $$\varepsilon (\omega ,\phi ,\theta ,\varphi )=\alpha (\omega ,\phi ,\theta ,\varphi ),$$

1b $$\varepsilon (\omega ,\phi ,\theta ,\varphi )=\frac{P(\omega ,\phi ,\theta ,\varphi ,T)}{{P}_{BB}(\omega ,\phi ,\theta ,\varphi ,T)},$$

1c $$\alpha (\lambda ,\phi ,\theta ,\varphi )=1-\rho (\omega ,\phi ,\theta ,\varphi )-\tau (\omega ,\phi ,\theta ,\varphi )=1-\sigma (\omega ,\phi ,\theta ,\varphi ),$$

where *σ* is the scattering, defined to take into account all reflected and transmitted light.

The comparative studies of absorption and thermal radiation spectra of three-valent rare-earth ions doped into a variety of oxide hosts have been undertaken in the first half of the 20^th^ century^50–52^. Thus, Wood^51^, using the Bausch and Lomb one-prism spectrograph and suitable color filters, has shown that in thin quartz rods doped with Nd^3+^ ions, the absorption bands (approximately) correspond to the thermal emission bands^50, 51^. Qualitatively similar behavior has been observed in pulverized and fused erbium oxide^50, 52^. Given the technology of the time, only the maxima of the spectral lines could be analyzed more or less accurately, but neither the line-shapes nor the line intensities. Correspondingly, the precise spectra of radiant emissivity *ε* could not be obtained and compared with the spectra of radiant absorptance *α*.

In this work, we study the spectra of absorptance *α*, thermal radiant power *P*, and emissivity *ε* of dielectric crystals doped with Er^3+^ ions (potential thermophotovoltaic converters), their agreement with the Kirchhoff’s law of thermal radiation and the limits of the law’s applicability.

## Results

### Model considerations

Let us assume that a crystal doped with rare-earth ions in high concentration has its maximal absorption coefficient in the visible-to-mid-infrared spectral range equal to ${k}_{abs}^{{\rm{\max }}}$ = 100 cm^−1^ and minimal absorption coefficient equal to ${k}_{abs}^{{\rm{\max }}}$ = 0.01 cm^−1^–typical values for rare-earth doped laser crystals^42, 53^. The absorption coefficient ${k}_{abs}^{{\rm{\max }}}$ = 100 cm^−1^ is too small to cause any significant change of the real part of the refractive index *η*, which, in the first approximation, can be assumed to be dispersion-less.

It is instructive to examine thermal emission and absorption occurring at three different length-scales.

1. When the size of the radiating body is large, $l\gg {({k}_{abs}^{{\rm{\min }}})}^{-1}$ = 1 m, then the body is opaque, *τ* → 0. Far from its edges, the body should have an appearance of an anthracite coal–black and moderately shiny. Following the Kirchhoff’s law (and easing the requirement of thermodynamic equilibrium), the emissive power *P* is predicted to be that of the blackbody *P*_*BB*_ multiplied by the (nearly spectrally independent) factor (1 − *ρ*),
  2 $$P={P}_{BB}(1-\rho ).$$
2. At the intermediate length-scale, ${({k}_{abs}^{{\rm{\min }}})}^{-1} > l\approx 1{\rm{cm}} > {({k}_{abs}^{{\rm{\max }}})}^{-1}$, the body is nearly opaque in the spectral range of strong absorption, $l > {({k}_{abs}^{{\rm{\max }}})}^{-1}$, and nearly translucent in the spectral range of small absorption, $l < {({k}_{abs}^{{\rm{\min }}})}^{-1}$. This length scale appears to be the most relevant to thermophotovoltaic devices based on rare-earth thermal converters. However, since the body is not completely opaque, it is questionable whether the Kirchhoff’s law, as formulated above, can be directly applied to this case as well as case 3 below. This gap of knowledge and lack of available working model motivated the present study of thermal radiation of not (completely) opaque bodies.
3. When the size of the body is small, $l\ll {({k}_{abs}^{{\rm{\max }}})}^{-1}$ = 100 μm, *α* → 0 and the body is translucent. Ignoring wave phenomena and considering light to be a stream of photons, the absorptance α(ω) is predicted to be equal to

3 $$\alpha (\omega )=1-\rho (\omega )-\tau (\omega )\approx {k}_{abs}(\omega )l$$

See section 1 of Supplementary Materials. (Note that applicability of the Kirchoff’s law to films has been demonstrated theoretically in ref. 54). Presuming that the Kirchhoff’s law can be extended to not opaque media and combining Eqs 1a and 3, one obtains

4 $$\varepsilon (\omega )=\alpha (\omega )\approx {k}_{abs}(\omega )l$$

**Figure 4:**
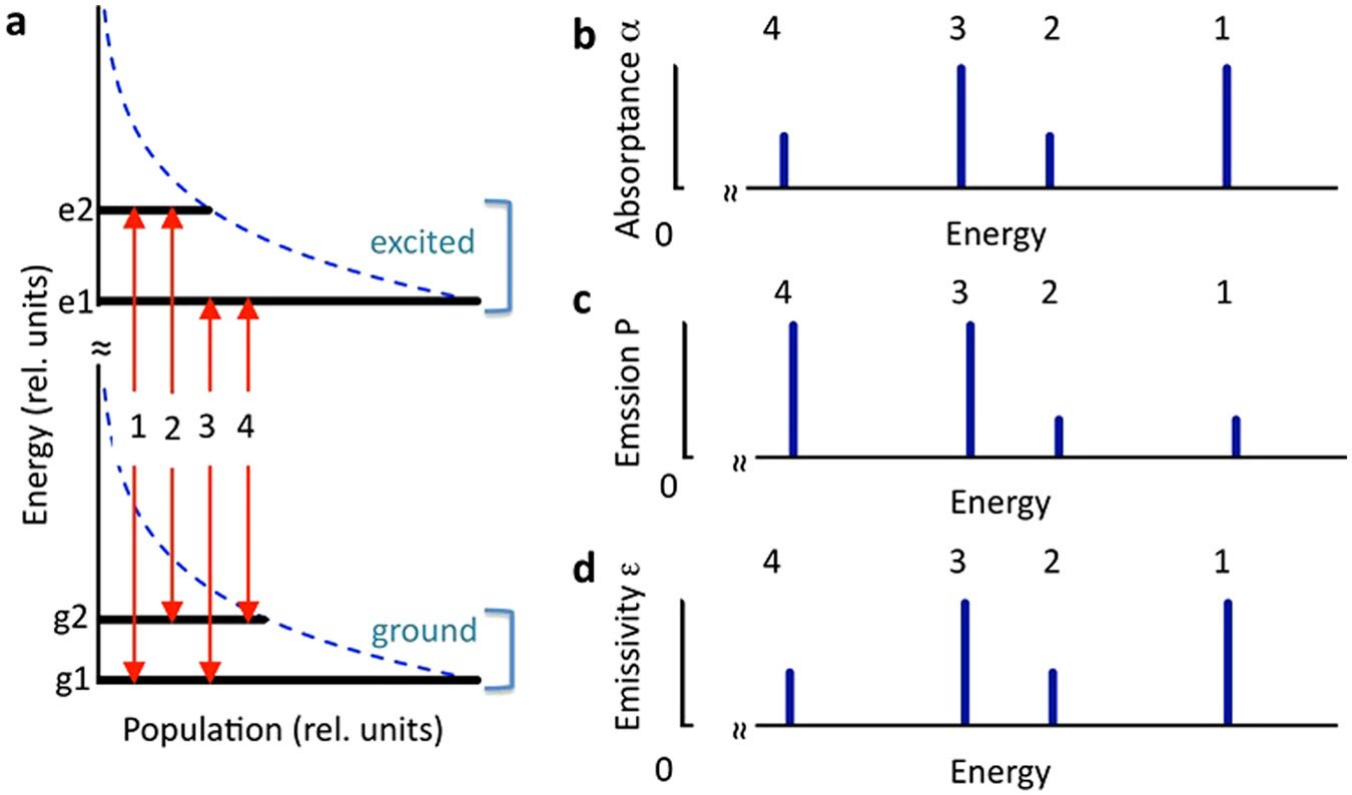
(**a**) Schematics of the energy states and Boltzmann distribution of population. Dashed line is the Boltzmann function. The population of the ground state components $|{g}_{1}\rangle$ and $|{g}_{2}\rangle$ is not in scale with the population of the excited state components $|{e}_{1}\rangle$ and $|{e}_{2}\rangle$. (**b**) Relative intensities of the absorptance spectral lines (*α* ∝ *k*_*abs*_). (**c**) Relative intensities of the emission spectral lines (radiant power *P*). (**d**) Relative intensities of emissivity spectral lines *ε*. (Same as for absorptance lines in Fig. 4b).

Note that the latter equation can be derived (see Supplementary Materials (Section 1)) using a simple spectroscopic model, which assumes that the spectra *k*_*abs*_(*ω*), *P*(*ω*) and *ε*(*ω*) are comprised of the four lines originating from the transitions between two Stark components of the ground state ($|{g}_{1}\rangle$ and $|{g}_{2}\rangle$) and two Stark components of the excited state ($|{e}_{1}\rangle$ and $|{e}_{2}\rangle$), Fig. 4a. If, for simplicity, all four transitions have the same spectral widths and cross sections, then the strengths of the absorption lines *k*_*abs*_(*ω*) are determined by Boltzmann populations of the ground state components $|{g}_{1}\rangle$ and $|{g}_{2}\rangle$ (Fig. 4b and Eq. S5). At the same time, the line intensities in the *very different* spectrum of emitted radiant power *P*(*ω*) are proportional to the Boltzmann populations of the excited state components $|{e}_{1}\rangle$ and $|{e}_{2}\rangle$ (Fig. 4c and Eq. S9).

However, according to the Kirchhoff’s law (Eq. 1), the spectrum of absorptance *α*(*ω*) should be compared not to the spectrum of radiant power *P*(*ω*) but rather to the spectrum of emissivity *ε*(*ω*) ≡ *P*(*ω*)/*P*_*BB*_(*ω*). The latter (Fig. 4d and Eq. S12) was shown to have exactly the same shape as the spectrum *α*(*ω*) (Fig. 4b and Eq. S9), see Supplementary Materials (Section 1).

The above mentioned derivation was based on the assumption that *k*_*abs*_ *l* ≪ 1 and no photons emitted deep in the volume of the medium are getting re-absorbed and re-emitted as they travel to the surface. In optically thick bodies, in the spectral ranges characterized by *k*_*abs*_ *l* ≥ 1, the photons emitted in the volume of the sample are re-absorbed and partly re-emitted by the medium. Therefore, the photons reaching the surface are emitted reasonably close to the surface (within $l\approx {k}_{abs}^{-1}$). At the same time, in the spectral ranges with *k*_*abs*_ *l* ≪ 1, the photons reaching the surface are generated uniformly throughout the whole volume of the body. This effect is expected to modify the radiation spectrum^55^ by suppressing the peaks and enhancing the wings of the emission spectral lines. The experimental studies of thermal radiation of dielectric crystals doped with Er^3+^ ions are presented below.

### Experimental results

Two samples used in the thermal radiation studies were Er doped Y_3_Al_5_O_12_, (80 at.%)Er:YAG, and Er doped LiYF_4_, (20 at.%)Er:YLF^56, 57^. The geometry of the samples and the details of the measurements are discussed in Methods.

#### Room temperature spectra

**Figure 5:**
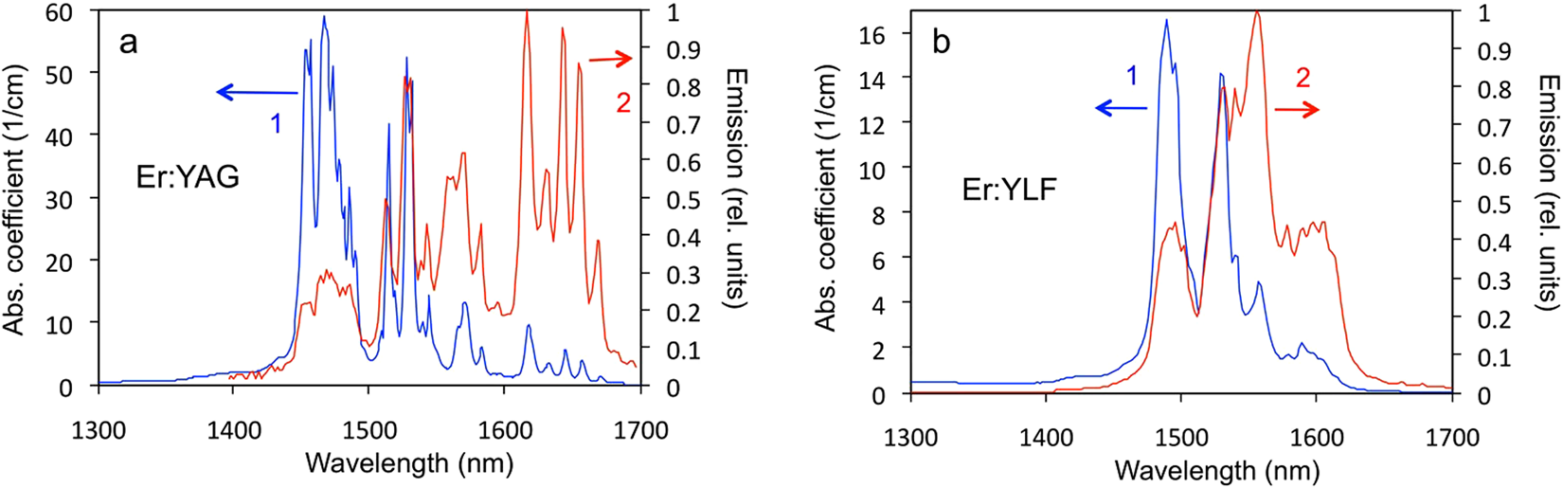
(**a**) Room temperature absorption (1) and emission (2) spectra of (80 at.%)Er:YAG (optical path *l* = 1.2 mm). (**b**) Same for (20 at.%)Er:YLF (optical path *l* = 3.0 mm).

The room temperature absorption and emission spectra of Er:YAG and Er:YLF are depicted in Fig. 5. In line with the arguments of Section 2.1 (see also Fig. 4 and Section 1 of Supplementary Materials), the distribution of the spectral line intensities in the absorption spectra are different from those in the emission spectra.

#### High temperature spectra

**Figure 6:**
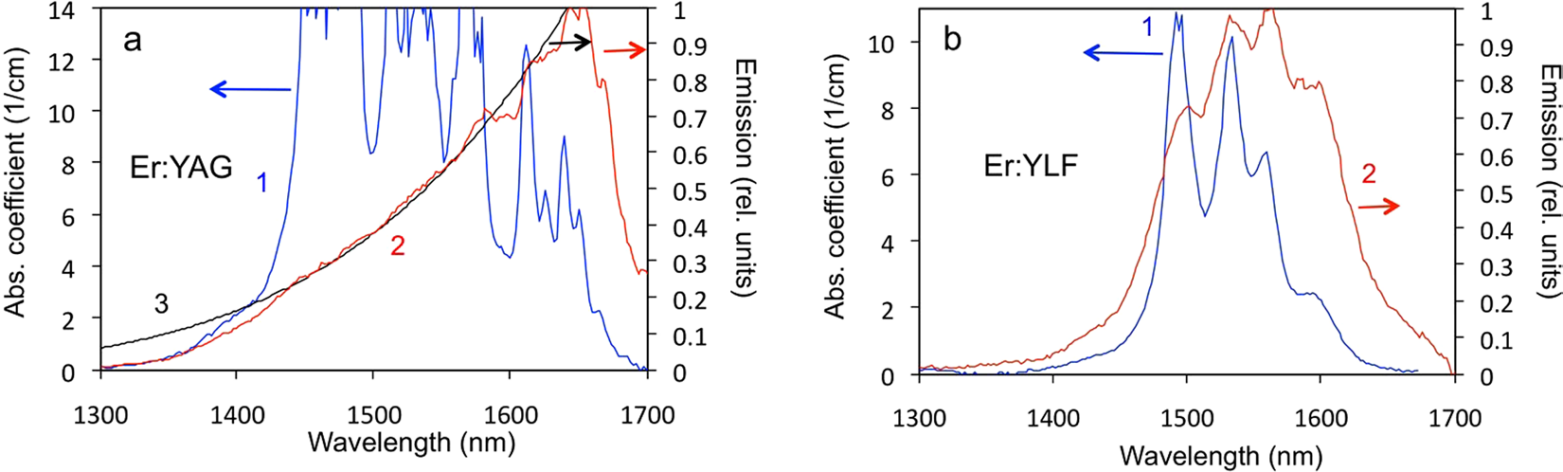
(**a**) High temperature (551 K) absorption spectrum (trace 1) and high temperature (583 K) emission spectrum (trace 2) of the (80 at.%)Er:YAG (optical path *l* = 3.8 mm). Black body emission spectrum calculated at the sample’s temperature 583 K (trace 3). Because of the large thickness of the crystal, the absorption coefficients in the peaks of the spectral lines (≥14 cm^−1^) could not be determined accurately (not shown). (**b**) High temperature (612 K) absorption spectrum (trace 1) and high temperature (588 K) emission spectrum (trace 2) of the (20 at.%)Er:YLF (optical path *l* = 3.0 mm).

**Figure 7:**
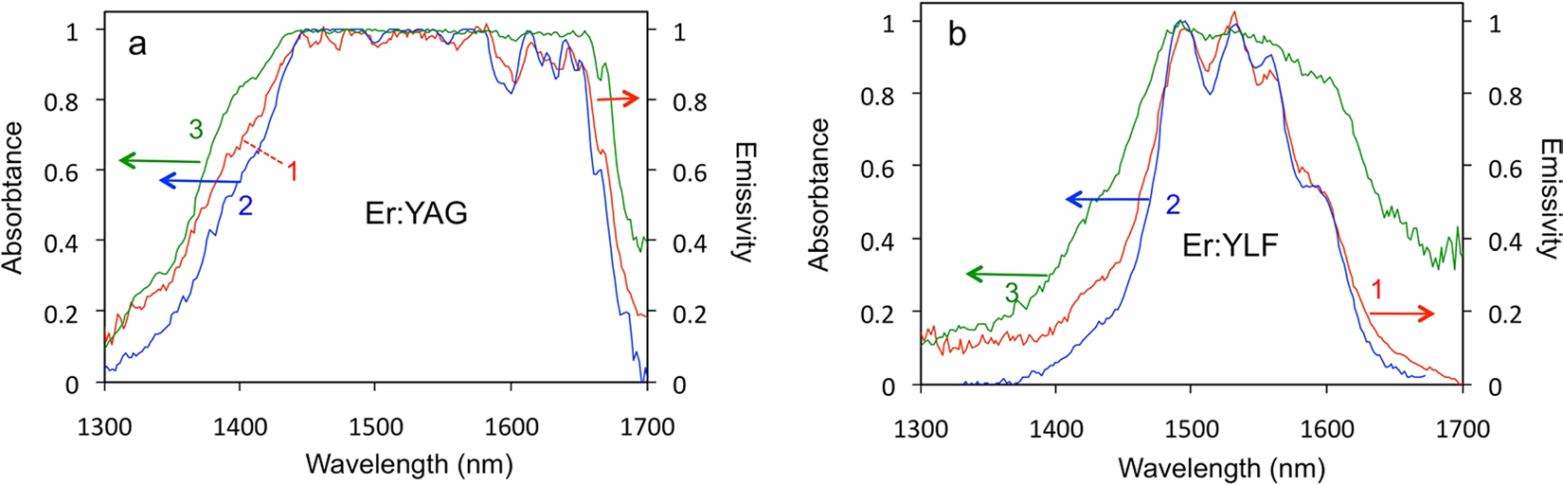
(**a**) Emissivity of the Er:YAG crystal (trace 1) and its absorptance measured in the transmission (trace 2) and scattering (trace 3) experiments. Traces 2 and 3 are normalized to unity. Trace 1 was scaled to have the best match with trace 2 between 1.44 μm and 1.65 μm. (**b**) Same for the Er:YLF crystal.

**Figure 8:**
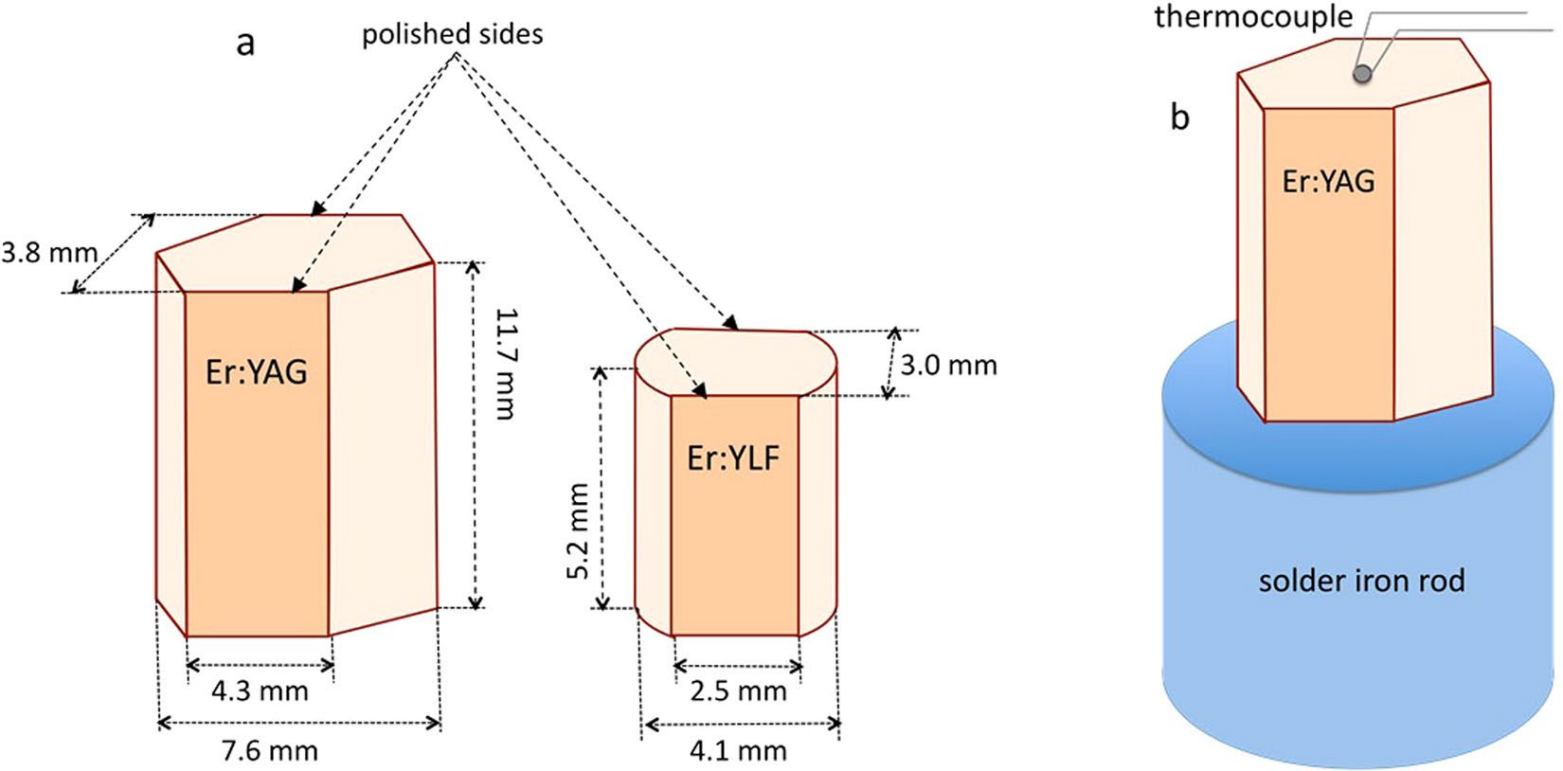
(**a**) Geometry of the Er^3+^ doped crystals used in the thermal radiation studies. (**b**) Crystal mounted on top of the solder iron rod (heater).

**Figure 9:**
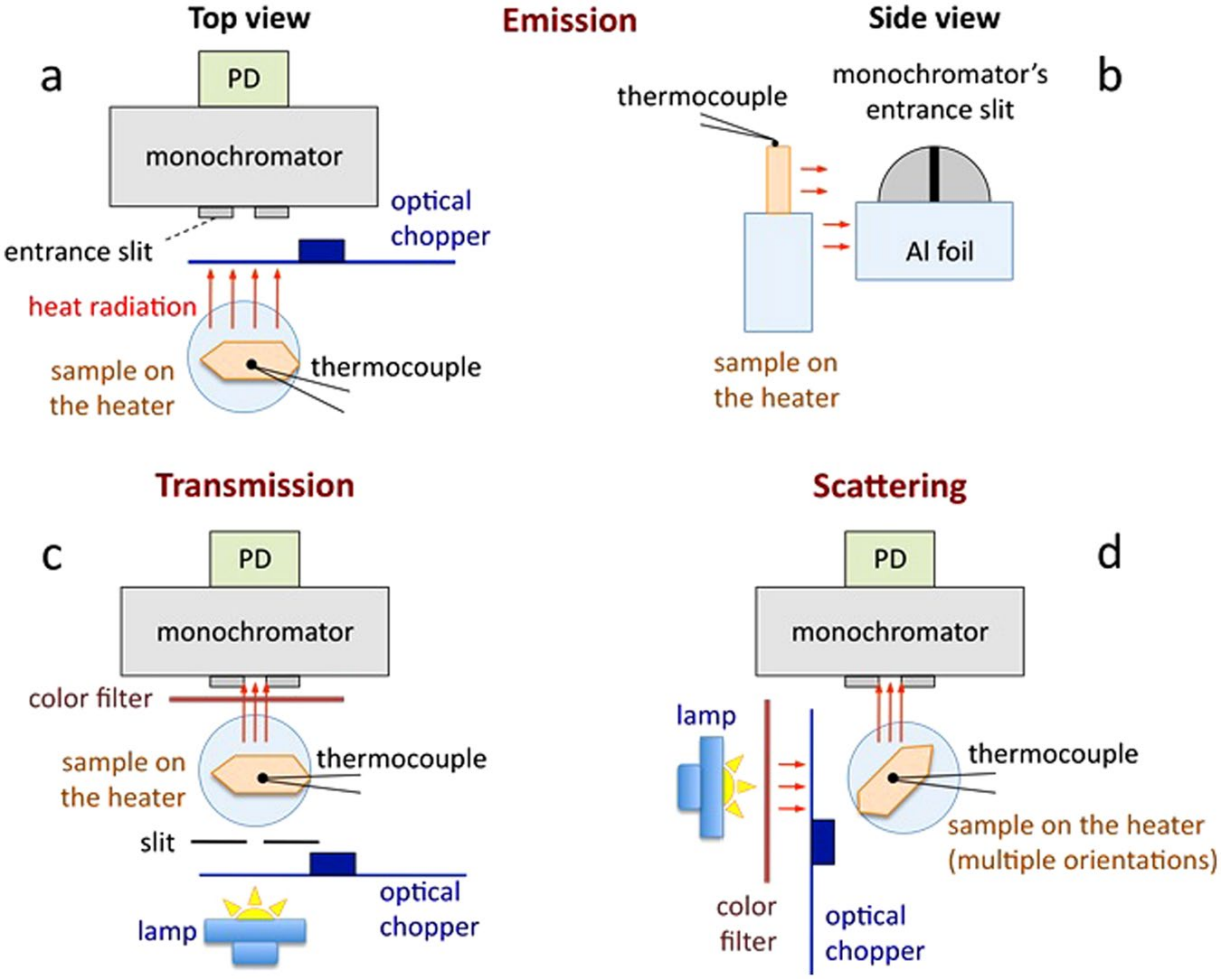
(**a**) Emission measurement setup (top view). (**b**) Sample on the heater stage in front of the entrance slit of the monochromator, which is partly covered with the aluminum foil in the emission measurements (side view). (**c**) Transmission measurement setup (top view). (**d**) Scattering measurement setup (top view).

The spectra of thermal radiation, transmittance and scattering of the heated Er:YAG and Er:YLF crystals have been acquired and used to derive the spectra of emissivity *ε*(*λ*) and absorptance *α*(*λ*) as described in Methods. The spectra of emitted radiant power *P*(*λ*) are shown as traces 2 in Fig. 6a and b, respectively, and the corresponding emissivity spectra *ε*(*λ*) are depicted as traces 1 in Fig. 7a and b. (As follows from the discussion of the experimental setups and the normalization procedure in Methods (see also Figs 8 and 9), only the shapes of the emissivity spectra, but not the absolute values *ε*(*λ)* can be analyzed in our experiment.)

The spectrum of absorption coefficients *k*_*abs*_(*λ*) (Fig. 6) as well as the spectrum of absorptance *α*(*λ*) (Fig. 7) have been acquired as described in Methods and Section 2 of Supplementary Materials.

In the Er:YAG crystal, multiple angular absorptance spectra *α*(*λ*, *θ*_*i*_, *φ*_*i*_, *θ*_*j*_, *ϕ*_*j*_) calculated (as discussed in Methods and Section 2 of Supplementary Materials) from the scattering spectra *σ*(*λ*, *θ*_*i*_, *φ*_*i*_, *θ*_*j*_, *ϕ*_*j*_), had nearly the same shape, Fig. 7a, trace 3. They were also reasonably close to the absorptance spectrum (Fig. 7a, trace 2) recorded in the transmission setup of Fig. 9c. In Er:YLF, the spectra *α*(*λ*, *θ*_*i*_, *φ*_*i*_, *θ*_*j*_, *ϕ*_*j*_) obtained from the scattering measurements carried out at different orientations of the sample (Fig. 7b, trace 3) were also reasonably similar to each other. However, none of them resembled the absorptance spectrum measured in the transmission experiments (Fig. 7b, trace 2). (Note that the experimental procedure discussed in Methods allowed one to deduce only the spectral shape of absorptance *α*(*λ*) but not its magnitude).

The spectra of Figs 5, 6 and 7 are discussed in detail in the section below.

## Discussion

### Trends in emission and absorption

In line with the arguments of Section 1 of Supplementary Materials (lower Stark components of the Er^3+^ ground state ^4^I_15/2_ and the excited state ^4^I_13/2_ are populated more than the higher energy components), the stronger absorption lines are expected in the short wavelength range of the ^4^I_15/2_–^4^I_13/2_ spectrum while the strongest emission lines are expected to be found at longer wavelengths. This is clearly the case of the Er:YAG and Er:YLF crystals, whose room temperature absorption and emission spectra are depicted in Fig. 5a and b.

With increase of the temperature, high-energy components of the ^4^I_15/2_ and ^4^I_13/2_ multifolds become more populated. In result, the “center of gravity” of the absorption spectrum is expected to shift toward longer wavelengths and that of the emission spectrum is supposed to shift toward shorter wavelengths. This trend is seen in the absorption and thermal radiation spectra of Er:YLF (Fig. 6b) and the absorption spectrum of Er:YAG (Fig. 6a). The behavior of the thermal radiation spectrum of Er:YAG is noteworthy. It does not show much of the blue shift, predicted based on the population arguments above. At the same time, in the broad spectral range, it closely follows the spectrum of the blackbody emission calculated at the same temperature, compare traces 2 and 3 in Fig. 6a. The significance of this behavior is emphasized in the section below.

The spectra of absorption (absorption coefficients) and emission (emitted power) in Figs 5 and 6 are not identical to each other and they should not be identical (see Section 1 of Supplementary Materials). According to the Kirchhoff’s law, the quantities, which have to be equal to each other, are the radiant absorptance *α*(*λ*) and the radiant emissivity *ε*(*λ*). The comparison of the spectral shapes of emissivity and absorptance, which carry important information on the applicability of the Kirchhoff’s law, is discussed below.

### Absorptance and emissivity of Er:YAG

The Er:YAG crystal used in our experiments has higher concentration of Er^3+^ ions and larger physical size than the Er:YLF crystal. It is nearly opaque and its transmittance is very close to zero between ~1.44 μm and ~1.65 μm, Fig. 7a. In the latter spectral range, the absorptance (evaluated in both transmission and scattering experiments) is spectrally flat and differentiates from unity by a small amount of nearly dispersion-less reflection. Remarkably, the shape of the absorptance spectrum *α*(*λ*) closely matches that of the emissivity *ε*(*λ*), which is also nearly flat and featureless. (The latter fact could be anticipated, since the emission spectrum of Er:YAG in Fig. 6a followed that of the blackbody at the same temperature). In this sense, at small angles of incidence, the crystal behaves *almost* like a blackbody (whose absorptance differentiates from unity by a small reflection coefficient *ρ*(*λ*)). We, thus, have shown that in the spectral range of the sample’s opacity, the absorptance *α*(*λ*) and emissivity *ε*(*λ*) are consistent with the predictions of the Kirchhoff’s law, even if the heated crystal is not in thermodynamic equilibrium with the room temperature environment. Outside of the opacity range, the agreement between the absorptance and the emissivity is poorer and depends on the geometry in which the absorptance is measured.

### Absorptance and emissivity of Er:YLF

The Er:YLF crystal was smaller and had lower concentration of Er^3+^ ions. It was not completely opaque. It had relatively large transmittance in the whole spectral range of interest, and its spectra of emissivity *ε*(*λ*) (Fig. 7b, trace 1) and absorptance *α*(*λ*), retrieved from the transmittance measurements (Fig. 7b, trace 2), featured spectral lines characteristic of Er^3+^ ions. (The latter were partly smeared out due to high temperature). Remarkably, the latter two traces had nearly similar shapes between 1.45 μm and 1.62 μm. This suggests that the Kirchhoff’s law is applicable to not opaque bodies as well. At the same time, the spectrum of absorptance deduced from a typical scattering experiment (Fig. 7b, trace 3) had a completely different shape. One can infer that the photons reaching the detector in the emission, transmission and scattering experiments experience different amounts of external and internal scattering off polished and unpolished surfaces of the sample. This can qualitatively explain the difference between traces 2 and 3 in Fig. 7b.

Note that the emission and absorption spectra in Fig. 6a and b were recorded at slightly different temperatures. Knowing the Stark splitting of the ground state multiplet ^4^I_15/2_ and the excited state multiplet ^4^I_13/2_ at the temperatures measured in the absorption and emission experiments, we estimate that the relative populations of the lowest and the highest Stark levels (and the intensities of the corresponding lines in the absorption and emission spectra) should vary with temperature by ~8% for YAG and ~3% for YLF. These small differences are practically not noticeable in the spectra of absorptance and emissivity in Fig. 7a and b. Moreover, the good agreement between the absorptance and the emissivity for both Er:YAG and Er:YLF serves as the evidence of adequately accurate temperature measurements.

### Use of Er^3+^ and other rare-earth ions in solar thermophotovoltaic applications

As dielectric crystals doped with Er^3+^ ions can emit thermal radiation in the relatively narrow near-infrared spectral band, the potential use of these and similar materials in solar thermophotovoltaic applications^34^ deserves serious consideration. We infer that thermophotovoltaic converters based on rare-earth doped materials should have the following desired properties.

(i) The material should be refractory (able to sustain high temperature).
(ii) The material should be opaque in both visible and near-infrared spectral ranges–to efficiently absorb solar energy and to emit thermal radiation. High concentration of Er^3+^ or other rare-earth dopant ions serves this purpose.
(iii) Even at large concentration of rare-earth ions, absorption in the visible spectral range may be too small. Co-doping the crystals with Cr^3+^ ions, which have strong broadband absorption in the visible part of the spectrum, as in *e.g*. yttrium scandium gallium garnet (Y_3_Sc_2_Ga_3_O_12_) doped with Er and Cr^41, 43^, should improve absorption of solar radiation significantly.
(iv) Thermal radiation band in the infrared range of the spectrum should be narrow. Therefore, the material should have weak crystal field and small Stark splitting of both ground and excited states^41^.
(v) Choosing the dopant ions, which have absorption and thermal radiation bands at longer wavelengths, such as Tm^3+^ (*λ* ≈ 1.8 μm), Ho^3+^ (*λ* ≈ 2.0 μm) and Dy^3+^ (*λ* ≈ 3.4 μm)^40^, can substantially decrease the operating temperature of the thermophotovoltaic converter and increase its efficiency.

Since not all the desired properties above may be available in one material, engineering of the thermophotovoltaic converter may require thorough optimization.

## Summary

The Kirchhoff’s law of thermal radiation, predicting that the radiant emissivity *ε* (the ratio of emitted radiant power to that from an ideal blackbody at the same temperature) should be equal to absorptance *α* (the ratio of absorbed power to incident power), is commonly formulated for opaque bodies in thermodynamic equilibrium with the environment. At the same time, many radiating bodies of practical importance are not opaque and not in thermodynamic equilibrium. The purpose of this work was to study the limits of applicability of the Kirchhoff’s law in an example of Er:YAG and Er:YLF dielectric crystals–potential radiation converters for thermophotovoltais applications.

Using a simple model, we have shown that the spectra of emissivity *ε* and absorptance *α* (at normal incidence) should have identical shapes in optically thin slabs at *k*_*abs*_ *l* ≪ l, without requirement of thermodynamic equilibrium with the environment (only thermalization within the ground state multifold and the excited state multifold is needed).

While one cannot claim a rigorous proof of the Kirchhoff’s law without absolute measurements, the equivalence of the spectral shapes of ε(λ) and α(λ) (e.g. in opaque bodies) is consistent with the predictions of the Kirchhoff’s law, while the difference in the spectral shapes of ε(λ) and α(λ) (e.g. in semi-translucent bodies) proves that the Kirchhoff’s law does not hold. Our experimental results, therefore, offer important information needed for understanding of the fundamental properties of thermal emission by opaque and not opaque bodies.

Experimentally, we have found the 3.8 mm thick (80 at.%) Er:YAG crystal to be nearly opaque between 1.44 μm and 1.65 μm. In this spectral range, its absorptance *α*(*λ*) is spectrally flat and differentiates from unity only by a small amount of reflection. One can say that at small angles of incidence, the crystal behaves almost like a blackbody. The shape of the emissivity spectrum *ε*(*λ*) closely matches that of absorptance *α*(*λ*), implying that the Kirchhoff’s law can adequately describe thermal radiation of opaque bodies, even if the requirement of thermodynamic equilibrium with the environment is not satisfied.

The (20 at.%) Er:YLF crystal had smaller size (3.0 mm thick), lower concentration of Er ions, and it was not opaque. Nevertheless, the spectrum of emissivity in this sample *ε*(*λ*) had almost the same shape as the spectrum of absorptance *α*(*λ*) derived from the transmission measurements. This suggests that the predictions of the Kirchhoff’s law can be extended to not opaque bodies, which are not in thermodynamic equilibrium with the environment. At the same time, the spectrum *α*(*λ*) retrieved from the scattering measurements had completely different shape. Therefore, in not opaque bodies, the spectra *α*(*λ*) and *ε*(*λ*) may coincide or not, depending on the geometry of the experiment as well as scattering-dependent photon residence time in the sample.

Lastly, rare-earth doped dielectric crystals appear to be promising radiation converters for thermophotovoltaic applications, which deserve further studies.

## Methods

### Experimental samples

Two samples used in the thermal radiation studies were Er doped Y_3_Al_5_O_12_ (Er:YAG) and Er doped LiYF_4_ (Er:YLF)^56, 57^. According to the manufacturer^56^, the concentration of Er^3+^ ions in the YAG crystal was equal to 80 at.%. This is consistent with the published spectroscopic data^42, 56^. The Er^3+^ concentration in the Er:YLF crystal, determined by comparing the absorption spectrum with the literature sources^57^, was equal to ~20 at.%. The shapes and sizes of the crystals are shown in Fig. 8a. The Er:YAG crystal was too thick to accurately measure the absorption coefficients in the maxima of the spectral lines. Therefore a thin, *l* = 1.2 mm, plate (with polished parallel sides) has been used in the spectrophotometer and spectrofluorimeter measurements as discussed below.

### Room temperature spectral measurements

Room temperature absorption spectra of the Er:YAG and Er:YLF crystals have been collected using Lambda 900 spectrophotometer equipped with the 150 mm integrating sphere (from Perkin Elmer). The spectra have been corrected for reflection. The room temperature emission spectra have been recorded using FluoroLog 3 Spectrofluorometer (from HORIBA Yobin Ivon).

### High temperature spectral measurements

#### Experimental setups

In the high-temperature spectral measurements, the crystals were mounted on a flat top of the solder iron rod (6 mm in diameter) wrapped with aluminum foil to reduce the thermal radiation, Fig. 8b. The rod was heated with the modified 40 W solder iron from Weller. The supplied voltage was controlled by the auto-transformer. A chromel-alumel thermocouple (K type, from OMEGA Engineering) was pressed against the top surface of the sample. In order to simulate the blackbody emitter, black soot was deposited onto the solder iron tip^58, 59^.

Three types of high temperature spectral measurements have been performed: emission, transmittance, and scattering. The corresponding experimental setups are shown in Fig. 9.

The optical spectra have been recorded using the MS257 monochromator (equipped with the 600 grooves/mm grating) and Merlin lock-in amplifier (from Newport/Oriel). The wavelength of the monochromator was calibrated using 632.8 nm line of the He-Ne laser, and the spectral response of the apparatus was calibrated using the spectrum of the black body (solder iron tip coated with black soot) recorded at the known temperature. The signal was detected with the peltier-cooled InGaAs photodiode (Electrical Optical Systems Inc.), whose spectral sensitivity range spanned from 0.95 μm to 1.75 μm. The 50 W halogen lamp served as the light source in the transmission and scattering measurements.

#### Emitted radiant power and emissivity

In the emission spectral measurements, the distance between the sample and the entrance slit of the monochromator was equal to ≈1 cm. The optical chopper (providing reference signal to the lock-in amplifier) was installed between the sample and the detector, Fig. 9a. In order to minimize the amount of thermal radiation of the solder iron rod (heater) getting to the entrance slit of the monochromator, the bottom half of the slit was covered with the aluminum foil, Fig. 9b. In the two consecutive measurements, we have recorded the spectrum of thermal radiation of (*i*) the sample on the heater stage and (*ii*) the heater stage without the sample. To obtain the thermal radiation spectrum of the sample without contribution from the heated environment, the (much weaker) latter signal was subtracted from the former one. When the resultant spectrum was divided by the response function of the apparatus, the spectrally corrected spectrum of the emitted radiant power *P* has been generated. To obtain the spectrum of radiant emissivity *ε*, the spectrum of emitted radiant power of the sample *P* was divided by that of the blackbody *P*_*BB*_ calculated at the same temperature.

Note that the thermal radiation spectra of Er doped samples and the black body were recorded in different geometries. (In the black body measurements, the solder iron tip covered with the black soot was oriented horizontally, the monochromator’s slit was not covered with aluminum foil, the size of the soldering iron rod was different from that of the sample, and the distance between the soldering iron rod and the slit of the monochromator was slightly different from that in the Er-doped sample measurements). Therefore, only the spectral shapes of the emissivity spectra *ε*(*λ*), but not the absolute emissivity values can be analyzed.

#### Transmission and absorption

The transmission spectral measurements were carried out using the setup depicted in Fig. 7c. The optical chopper was installed between the lamp and the crystal. Therefore, thermal radiation of the sample did not contribute to the output signal of the lock-in amplifier. The external slit installed several millimeters away from the sample ensured that no photons emitted by the lamp reached the entrance slit of the monochromator bypassing the crystal. The long-pass color filter blocked the (second order of diffraction) visible light, which otherwise would mix with the (first order of diffraction) infrared light to be studied. In a typical experiment, two spectra have been recorded, with and without the sample. The presence of the crystal slightly reshaped the light beam (possibly due to unintentional curving of the nominally flat parallel polished crystal surfaces, which produced the lensing effect). Therefore, after dividing the former spectrum by the latter, the resultant transmittance spectrum was further multiplied by a correction factor, bringing the transmission in the spectral ranges, which did not have any absorption, to 100%.

#### Scattering

In the scattering measurements, the crystal was installed in front of the entrance slit of the monochromator and illuminated from the side, Fig. 7d. The optical chopper was installed between the lamp and the crystal. Therefore, the thermal radiation of the sample did not contribute to the scattering (or the reflection) spectrum. The long-pass color filter was used to block the visible light. In multiple measurements, the crystal was positioned at various angles relative to the lamp (*θ*_*i*_, *ϕ*_*i*_) and the monochromator’s slit (*θ*_*j*_, *ϕ*_*j*_) and was turned to them with its polished or unpolished sides. Correspondingly, in different particular measurements, the specular reflected light and the diffused light reaching the detector were mixed in different proportions and the photons experienced larger or smaller number of scattering events before they exited the samples.

In the control measurement, the crystal was replaced with the broadband white diffused reflector (from Lab Sphere). Experimentally, the two spectra were collected, one with the crystal and one with the diffused reflector. Then the former was divided by the latter, and the resultant scattering spectrum was scaled to reach unity in the spectral ranges where the crystals did not have any absorption (as it is explained above and in Section 2 of Supplementary Materials). This angular-dependent scattering spectrum *σ*(*λ*, *θ*_*i*_, *φ*_*i*_, *θ*_*j*_, *ϕ*_*j*_) was used to obtain the spectrum of the angular sample’s absorptance for given directions of illumination and data collection, *α*(*λ*, *θ*_*i*_, *φ*_*i*_, *θ*_*j*_, *ϕ*_*j*_) = 1 − *σ*(*λ*, *θ*_*i*_, *φ*_*i*_, *θ*_*j*_, *ϕ*_*j*_). (This procedure allowed one to deduce only the spectral shape of absorptance but not its magnitude)^60–63^.

## Electronic supplementary material

Supplementary Materials

## References

[1] Da Rosa, A. Fundamentals of Renewable Energy Processes (Academic Press, 2012).

[2] Boyle, G. Renewable Energy: Power for a Sustainable Future (Oxford University Press, 2012).

[3] Kirk, A. P. Solar Photovoltaic Cells: Photons to Electricity (Academic Press, 2014).

[4] Shockley W, Queisser HJ (1961). Detailed Balance Limit of Efficiency of p‐n Junction Solar Cells. J. Appl. Phys..

[5] Streetman, B. G. & Banerjee, S. *Solid State electronic Devices* (Prentice Hall, 2000).

[6] Henry CH (1980). Limiting efficiencies of ideal single and multiple energy gap terrestrial solar cells. J. Appl. Phys..

[7] De Vos A (1980). Detailed balance limit of the efficiency of tandem solar cells. J. Phys. D.

[8] Marti A, Araujo A (1996). Limiting efficiencies for photovoltaic energy conversion in multigap systems. Sol. Energ. Mat. Sol. Cells.

[9] Brown AS, Green MA (2002). Detailed balance limit for the series constrained two terminal tandem solar cell. Phys. E.

[10] Ruppel W, Wurfel P (1980). Upper limit for the conversion of solar energy. IEEE Trans. Electron Devices.

[11] Swanson RM (1979). A proposed thermophotovoltaic solar energy conversion system. in Proc. IEEE.

[12] Spirkl W, Ries H (1985). Solar thermophotovoltaics: An assessment. J. Appl. Phys..

[13] Landsberg PT, Baruch P (1989). The thermodynamics of the conversion of radiation energy for photovoltaics. J. Phys. Math. Gen..

[14] Badescu V (2001). Thermodynamic Theory of Thermophotovoltaic Solar Energy Conversion. J. Appl. Phys..

[15] Badescu V (2005). Upper bounds for solar thermophotovoltaic efficiency. Renew. Energy.

[16] Tobias I, Luque A (2002). Ideal efficiency and potential of solar thermophotonic converters under optically and thermally concentrated power flux. IEEE Trans. Electron Devices.

[17] Harder NP, Würfel P (2003). Theoretical limits of thermophotovoltaic solar energy conversion. Semicond. Sci. Technol..

[18] Rephaeli E, Fan S (2009). Absorber and emitter for solar thermo-photovoltaic systems to achieve efficiency exceeding the Shockley-Queisser limit. Optics Express.

[19] DeSutter J, Bernadi MP, Francouer M (2016). Determination of thermal emission spectra maximizing thermophotovoltaic performance using a generic algorithm. Energy Conversion and Management.

[20] Heinzel A (2000). Radiation filters and emitters for the NIR based on periodically structured metal surfaces. J. Mod. Opt..

[21] Sai H, Yugami H (2004). Thermophotovoltaic generation with selective radiators based on tungsten surface gratings. Appl. Phys. Lett..

[22] Celanovic I, Jovanovic N, Kassakian J (2008). Two-dimensional tungsten photonic crystals as selective thermal emitters. Appl. Phys. Lett..

[23] Chen YB, Zhang ZM (2007). Design of tungsten complex gratings for thermophotovoltaic radiators. Opt. Commun..

[24] Lenert A (2014). Nanophotonic Solar Thermophotovoltaic Device. Nature Nanotechnology.

[25] Chaudhuri TK (1992). A solar thermophotovoltaic converter using Pbs photovoltaic cells. Int. J. Energy Res..

[26] Gee, J. M., Moreno, J. B., Lin, S.Y. & Fleming, J. G. Selective emitters using photonic crystals for thermophotovoltaic energy conversion, in Conference Record of the *Twenty-Ninth IEEE Photovolt. Spec. Conf*., Piscataway, NJ (2002).

[27] Tikhodeev SG (2002). Quasiguided modes and optical properties of photonic crystal slabs. Phys. Rev. B.

[28] Lin SY, Moreno J, Fleming JG (2003). Three-dimensional photonic-crystal emitter for thermal photovoltaic power generation. Appl. Phys. Lett..

[29] Andreev, V. M. *et al*. Solar thermophotovoltaic system with high temperature tungsten emitter, *Conference Record of the Thirty-first IEEE Photovoltaic Specialists Conference, Lake Buena Vista, FL, USA, 2005*, pp. 671–674.

[30] Celanovic I, Perreault D, Kassakian J (2005). Resonant-cavity enhanced thermal emission. Phys. Rev. B.

[31] Chan DL, Soljacić M, Joannopoulos JD (2006). Thermal emission and design in 2D-periodic metallic photonic crystal slabs. Opt. Express.

[32] Vlasov, A. S. *et al*. TPV Systems with Solar Powered Tungsten Emitters, *in AIP Conference Proceedings 890, Spain, 2006*, (AIP Publishing 2007), pp. 327–334.

[33] Rephaeli E, Fan S (2008). Tungsten black absorber for solar light with wide angular operation range. Appl. Phys. Lett..

[34] Yugami, H. *et al*. Solar thermophotovoltaic using Al_2_O_3_/Er_3_Al_5_O_12_ eutectic composite selective emitter, *IEEE Photovoltaic Spec*. *Conf*. *28*, *Anchorage*, *2000*, *pp*. *1214*–*1217*.

[35] Guazzoni GE (1971). High-Temperature Spectral Emittance of Oxides of Erbium, Samarium, Neodymium and Ytterbium. Appl. Spectroscopy.

[36] Ferrari, C. *et al*. Overview and Status of Thermophotovoltaic Systems, 68^th^ Conference of the Italian Thermal Machines Engineering Association, ATI2013; Energy Procedia **45** 160–169 (2014).

[37] Chubb, D. L., Pal, A. T., Patton, M. O., & Jenkins, P. P. Rare earth doped yttrium aluminum garnet (YAG) selective emitters, (1998) (Date of access: 06/01/2015).

[38] Chubb DL, Pal AT, Patton MO, Jenkins PP (1999). Rare-earth doped high-temperature ceramic selective emitters. Journal of the European Ceramic Society.

[39] Ferguson LG, Dogan F (2002). Spectral analysis of transition metal-doped MgO “matched emitters” for thermophotovoltaic energy conversion. J. Mater. Science.

[40] Pressley, R. J. Handbook of Lasers with Selected Data on Optical Technology (Weast, R. C. *et al*.) Ch. 13, pp. 371–417 (CRC Press 1971).

[41] Kaminskii, A. A. *Crystalline lasers: Physical processes and operating schemes*, pp. 176,187 (CRC press, New York 1996).

[42] Scientific Materials Corp, Material properties of 50% Er: YAG: http://www.scientificmaterials.com/products/er-yag.php (Date of access: 06/01/2015).

[43] Zharikov EV (1986). Spectral luminescence, and lasing properties of a yttrium scandium gallium garnet crystal activated with chromium and erbium. Soviet Journal of Quantum Electronics.

[44] Johnson LF, Geusic JE, Van Uitert LG (1965). Coherent Oscillations from Tm^3+^, Ho^3+^, Yb^3+^ and Er^3+^ ions in Yttrium Aluminum Garnet. Appl. Phys. Lett..

[45] Kirchhoff, G. R. On the relation between the emissive and the absorptive power of absorptive power of bodies for heat and light, *Gesammelte Abhandlungen*, pp. 571–598, Leipzig, 1882. (Date accessed: 01/01/2015).

[46] Landau, L. D. & Lifshitz, E. M. *Statistical Physics, Second Revised and Enlarged Edition, Course of Theoretical Physics* Vol. 5 (translated by J.B. Sykes and M.J. Kearsley.) (Pergamon Press, 1958).

[47] Nicodemus FE (1965). Directional Reflectance and Emissivity of an Opaque Surface. Appl. Opt..

[48] Greffet JJ, Nieto-Vesperinas M (1998). Field theory for generalized bidirectional reflectivity: derivation of Helmholtz’s reciprocity principle and Kirchhoff’s law. J. Opt. Soc. Am. A.

[49] Snyder WC, Wan Z, Li X (1998). Thermodynamic constraints on reflectance reciprocity and Kirchhoff’s law. Appl. Optics.

[50] Anderson JA (1907). Astrophysical Journal. An International Review of Spectroscopy and Astronomical Physics.

[51] Wood RW (1931). Selective radiation of colored and pure fused quartz. Phys. Rev.

[52] Wood, R. W. *Physical Optics* (Wood, R. W.) pp. 787–789 (Capital City Press Inc., 1934).

[53] Innocenzi ME (1990). Optical absorption in undoped yttrium aluminum garnet. J. Appl. Phys..

[54] Edalatpour S, Francoeur M (2013). Size Effect on the emissivity of thin films. Journal of Quantitative Spectroscopy & Radiative Transfer.

[55] Oelkrug, D. *Topics in Fluorescence Spectroscopy: Vol. 4: Probe Design and Chemical Sensing*, (Lakowicz, J. R.) Ch. 8: p. 223 (Kluwer Academic Publishers, New York, 1994).

[56] Absorption coefficient of 50% Er: YAG http://www.northropgrumman.com/BusinessVentures/SYNOPTICS/Products/LaserCrystals/Pages/ErYAG.aspx (Date of access: 01/01/2015).

[57] Dergachev, A. & Moulton, P. F. Advanced Solid-State Photonics (ed. Zayhowski, J.), *Tunable CW Er:YLF Diode-Pumped Laser* (Optical Society of America, 2003), paper 3. https://www.researchgate.net/publication/228940082_Semiconductor_Q-switched_Short-Pulse_High-Power_MHz-Rate_Laser (Date of access: 01/01/2015).

[58] Kats MA (2013). Vanadium dioxide as a natural disordered metamaterial: perfect thermal emission and large broadband negative differential thermal emittance. Phys. Rev. X.

[59] Noginov MA (2015). Thermal radiation of lamellar metal-dielectric metamaterials and metallic surfaces. Optical Material Express.

[60] Siegman, A. E. *Lasers* (Kelly, A.) p. 1283 (University Science Books, 1986).

[61] Koechner, W. & Bass, M. *Solid State Lasers: a graduate text* (eds Koechner, W. & Bass, M.) p. 409 (Springer, New York, 2003).

[62] Sivukhin, D. V. General course of physics, Vol. 4, Optics (in Russian).

[63] Pantell, R. H. & Puthoff, H. E. *Fundamentals of Quantum Electronics* (John Wiley & Sons Inc., 1969).

