# Supplementary material for: Thermal radiation of Er doped dielectric crystals: Probing the range of applicability of the Kirchhoff’s law
Source: Sci Rep. 2017 May 17;7:2040. doi: 10.1038/s41598-017-01544-3 (PMC5435707; doi:10.1038/s41598-017-01544-3)
Supplement: Supplementary file 1 — Supplementary Materials [file 41598_2017_1544_MOESM1_ESM.pdf]

Thermal radiation of Er doped dielectric crystals: Probing the range of applicability of the Kirchhoff's law

Ekembu K. Tanyi [1], Brandi T. Burton [1], Evgenii E. Narimanov [2], and M. A. Noginov [1*].
[1] *Center for Materials Research, Norfolk State University, Norfolk, VA 23504*
[2] *Birck Nanotechnology Center, Department of Electrical and Computer Engineering, Purdue University, West Lafayette, IN 47907, USA*
[*] *mnoginov@nsu.edu*

SUPPLEMENTARY MATERIALS

S1. ABSORPTANCE AND EMISSIVITY

S1.1 Reflectance, transmittance, and absorptance

Let us consider the simplest situation when light falls, at normal incidence, onto a slab of material with two parallel ideally polished walls. Ignoring wave phenomena and considering light to be a stream of photons, the reflectance ρ and transmittance τ are described by the following formula,

$$\rho = r + \frac{(1-r)^2 r \exp(-2k_{abs}l)}{1 - r^2 \exp(-2k_{abs}l)}, \qquad \text{(S1)}$$

$$\tau = \frac{(1-r)^2 \exp(-k_{abs}l)}{1 - r^2 \exp(-2k_{abs}l)}, \qquad \text{(S2)}$$

where ρ is the reflectance at the medium/air interface. It is easy to show that at $k_{abs}l << 1$,

$$\alpha(\omega) = 1 - \rho(\omega) - \tau(\omega) \approx k_{abs}(\omega)l. \qquad \text{(3)}$$

The question arises: what are the expected spectra of thermal radiation power *P(ω)* and thermal emissivity *ε(ω)* if the Kirchhoff's laws can be extended to not opaque media? In this case, combining Eqs. 1a and 3, one obtains

$$\varepsilon(\omega) = \alpha(\omega) = k_{abs}(\omega)l \qquad \text{(4).}$$

Alternatively, this equation can be derived using simple spectroscopic considerations as described below.

S1.2 Absorptance of translucent samples

For simplicity, let us assume that the spectrum $k_{abs}(\omega)$ is comprised of the four lines originating from the transitions between two Stark components of the ground state ($|g_1\rangle$ and $|g_2\rangle$) and two Stark components of the (metastable) excited state ($|e_1\rangle$ and $|e_2\rangle$), Fig. 4a. Correspondingly,

$$\begin{aligned}&\alpha(\omega) \propto k_{abs}(\omega)\\ &= \sigma_1(\Omega_1,\omega)m_{g_1} + \sigma_2(\Omega_2,\omega)m_{g_2},\\ &+\sigma_3(\Omega_3,\omega)m_{g_1} + \sigma_4(\Omega_4,\omega)m_{g_2}\end{aligned} \qquad \text{(S3)}$$

where $\sigma_i(\Omega_i,\omega)$ are the absorption cross section spectra of the four transitions with the maxima at Ω_i (Fig. 4a), and m_{g_1} and m_{g_2} are the population number densities of the ground state components $|g_1\rangle$ and $|g_2\rangle$.

Let us also assume, for simplicity, that the four transitions have identical line-shapes and cross sections ($\sigma = \sigma_{1(\omega=\Omega_1)} = \sigma_{2(\omega=\Omega_2)} = \sigma_{3(\omega=\Omega_3)} = \sigma_{4(\omega=\Omega_4)}$). In this case, the intensities of the four absorption lines are solely determined by the populations m_{g_1} and m_{g_2}, which are governed by the Boltzmann statistics[60,61]

$$\frac{m_i}{m_j} = \exp\left(-\frac{\Delta E_{ij}}{k_B T}\right). \qquad \text{(S4)}$$

Here ΔE_{ij} is the energy difference between states $|i\rangle$ and $|j\rangle$, T is the temperature, k_B is the Boltzmann constant, and no degeneracy is assumed. Substituting Eq. S4 to Eq. S3, one obtains

$$\alpha(\omega) \propto k_{abs}(\omega)$$
$$\propto \sigma(\Omega_1,\omega)+\sigma(\Omega_2,\omega)\exp\left(-\frac{\Delta E_{g_1\to g_2}}{k_B T}\right) \quad . \qquad \text{(S5)}$$
$$+\sigma(\Omega_3,\omega)+\sigma(\Omega_4,\omega)\exp\left(-\frac{\Delta E_{g_1\to g_2}}{k_B T}\right)$$

The corresponding strengths of the absorption lines are schematically shown in Fig. 4b. In accord with Eq. S5 and the arguments above, the absorption transitions originating from the lower state $|g_1\rangle$ (lines 1 and 3) have larger strengths than those originating from the higher state $|g_2\rangle$ (lines 2 and 4). Therefore, if the Kirchhoff's law is applicable to not opaque bodies ($k_{abs}l \ll 1$), then the spectrum of radiative emissivity $\varepsilon(\omega)$ should resemble that of Fig. 4b.

S1.3 Emitted radiant power and emissivity of translucent samples

Returning to the arguments of thermal radiation, light emission originates from atomic and molecular transitions between the higher energy states and the lower energy states. The only difference between thermal radiation and other types of emission, *e.g.* luminescence or spontaneous emission, is the method of excitation of the higher energy states – in the case of thermal radiation, it occurs as the result of thermal motion of atoms or molecules[62].

Treating thermal radiation as a thermally excited spontaneous emission, one would expect the spectrum of radiant power (emitted in volume V and frequency interval $d\omega$) to be given by

$$P(\omega)/V = [A_1 g(\Omega_1,\omega) m_{e_2} + A_2 g(\Omega_2,\omega) m_{e_2} + A_3 g(\Omega_3,\omega) m_{e_1} + A_4 g(\Omega_4,\omega) m_{e_1}]\hbar\omega d\omega \quad , \qquad \text{(S6)}$$

where A_i are the spontaneous emission rates of the transitions depicted in Fig. 4a, $g(\Omega_i,\omega)$ are the transitions' line-shapes, Ω_i are the transitions' central frequencies, m_{e_1} and m_{e_2} are the population number densities of the excited state components $|e_1\rangle$ and $|e_2\rangle$, and $\hbar$ is the Planck's constant. Since the spontaneous emission rate is related to the emission cross section as[63]

$$A_i g(\Omega_i,\omega) = \sigma_i(\Omega_i,\omega)\frac{\omega^2\eta^2}{\pi^2 c^2} \quad , \qquad \text{(S7)}$$

Eq. (S7) can be re-written as

$$P(\omega)/V = [\sigma_1(\Omega_1,\omega) m_{e_2} + \sigma_2(\Omega_2,\omega) m_{e_2} + \sigma_3(\Omega_3,\omega) m_{e_1} + \sigma_4(\Omega_4,\omega) m_{e_1}]\frac{\hbar\omega^3\eta^2}{\pi^2 c^2} d\omega \qquad \text{(S8)}$$

(where η is the refractive index). If all emission spectral lines have identical shapes and cross sections, then the intensities of the emission lines will be determined by the excited state concentrations m_{e_1} and m_{e_2} (whose ratio is different from that of m_{g_1} and m_{g_2}) and ω^3. Whether the excited state is in thermal equilibrium with the ground state (as in the case of thermal radiation) or not (as in the case of optically pumped spontaneous emission), the ratio of the concentrations m_{e_1} and m_{e_2} is determined by Eq. (S4). Therefore,

$$\frac{P(\omega)}{V} = m_{e_1}[\sigma(\Omega_1,\omega)\exp\left(-\frac{\Delta E_{e_1\to e_2}}{k_B T}\right) + \sigma(\Omega_2,\omega)\exp\left(-\frac{\Delta E_{e_1\to e_2}}{k_B T}\right) + \sigma(\Omega_3,\omega) + \sigma(\Omega_4,\omega)]\frac{\hbar\omega^3\eta^2}{\pi^2 c^2} d\omega$$

(S9)

Correspondingly, the ratios of the spectral line intensities in the emission spectra (Fig. 4c) are distinctly different from those in the absorption spectra (Fig. 4b). In Fig. 4c, it was assumed that

$(\Omega_1 - \Omega_4) << \Omega_1, \Omega_4$. Therefore, the ω^3 dependence as well as the frequency dependence of the refractive index η could be neglected. (The agreement between the absorption and the emission spectra did not improve if the relatively weak dependences above were taken into account.)

However, according to the Kirchhoff's law (Eq. 1), the spectrum of absorptance $\alpha(\omega)$ should be compared not to the spectrum of radiant power $P(\omega)$ but rather to the spectrum of emissivity $\varepsilon(\omega) \equiv P(\omega)/P_{BB}(\omega)$. As the spectral radiance of a blackbody (radiant energy per unit time, unit projected area, unit solid angle and frequency interval $d\omega$) is given by[63]

$$P_{BB}(\omega) = \frac{\hbar\omega^3}{4\pi^3 c^2} \frac{1}{\exp(\hbar\omega/k_B T) - 1}, \qquad \text{(S10)}$$

the emissivity is proportional to

$$\varepsilon(\omega) \propto [\sigma(\Omega_1,\omega)\exp\left(-\frac{\Delta E_{e_1\to e_2}}{k_B T}\right) + \sigma(\Omega_2,\omega)\exp\left(-\frac{\Delta E_{e_1\to e_2}}{k_B T}\right)$$
$$+\sigma(\Omega_3,\omega) + \sigma(\Omega_4,\omega)]\eta^2[\exp(\hbar\omega/k_B T) - 1]$$

(S11)

At $\hbar\Omega_i >> k_B T$, small widths of the spectral lines $\delta\omega_i < \Omega_i$, and spectrally independent refractive index η,

$$\varepsilon(\omega) \propto$$
$$\propto \sigma(\Omega_1,\omega)\exp\left(\frac{E_{\Omega_1}}{k_B T} - \frac{\Delta E_{e_1\to e_2}}{k_B T}\right) + \sigma(\Omega_3,\omega)\exp\left(\frac{E_{\Omega_3}}{k_B T}\right)$$
$$+\sigma(\Omega_2,\omega)\exp\left(\frac{E_{\Omega_2}}{k_B T} - \frac{\Delta E_{e_1\to e_2}}{k_B T}\right) + \sigma(\Omega_4,\omega)\exp\left(\frac{E_{\Omega_4}}{k_B T}\right) \text{(S12)}$$
$$\propto \sigma(\Omega_1,\omega) + \sigma(\Omega_2,\omega)\exp\left(-\frac{\Delta E_{g_1\to g_2}}{k_B T}\right) + \sigma(\Omega_3,\omega)$$
$$+\sigma(\Omega_4,\omega)\exp\left(-\frac{\Delta E_{g_1\to g_2}}{k_B T}\right)$$

(since $E_{\Omega_2} - \Delta E_{e_1\to e_2} = E_{\Omega_4}$, $E_{\Omega_4} + \Delta E_{g_1\to g_2} = E_{\Omega_3}$, and $E_{\Omega_1} - \Delta E_{e_1\to e_2} = E_{\Omega_3}$, Fig. 4a), which is identical to the spectral dependence of the absorption coefficient $k_{abs}(\omega)$ and absorptance $\alpha(\omega)$ (compare Eq. S5 and Figs. 4b and 4d).

Therefore, in a simple example, we have demonstrated that the Kirchhoff's law of thermal radiation $\varepsilon(\omega) \equiv P(\omega)/P_{BB}(\omega) \propto \alpha(\omega)$ can be applied to optically thin bodies, $k_{abs}l$<<1. Although, by no means, does this derivation serves as a rigorous proof applicable to all not opaque bodies, it suggests that the Kirchhoff's law can likely be applied to a variety of bodies with different degrees of opaqueness, such as doped crystals and glasses.

S2. ABSORPTANCE OF A SLAB AT NORMAL INCIDENCE ANGLE

As follows from Eqs. 1c, S1 and S2, at normal incidence of light onto a slab with parallel polished walls, the absorption coefficients $k_{abs}(\lambda)$ and the absorptance $\alpha(\lambda)$ can be determined precisely only if both transmittance $\tau(\lambda)$ and reflectance $\rho(\lambda)$ spectra are known. In practice, transmittance spectra are routinely measured in standard (*e.g.* spectrophotometer) experiments, while the samples' reflectance is available much more rarely. Here we show that for the absorption strengths and physical sizes characteristic of our Er doped crystals, the *approximate* method described below, based on knowledge of the transmittance spectra $\tau(\lambda)$ and the refractive index η, can be used to determine k_{abs} and α with high degree of accuracy.

In the example below, let us assume that the *exact* absorption spectrum $k^e_{abs}(\lambda)$ (given by *e.g.* trace 1 in Fig. S1a) and the refractive index η=1.806 (which is assumed to be constant in the whole range of interest, 1.3 μm to 1.7 μm) are known and that the absorption lines are too weak to affect the sample's normal incidence reflectance r=0.0825 at the single air/medium interface. (For the purpose of this

discussion, $k^e_{abs}(\lambda)$ can be any plausibly looking spectrum characteristic of the samples studied.) Then, using Eqs. S1, S2 and 1c, we can model the *exact* spectra $\tau^e(\lambda)$, $\rho^e(\lambda)$ and $\alpha^e(\lambda)$, Fig. S1b. (In calculations, l was equal to 3.8 mm.)

In order to simulate our approximate retrieval procedure, we will further multiply the spectrum $\tau^e(\lambda)$ by a certain scaling factor x to make the corrected transmittance τ^c to be equal to unity in the spectral ranges that do not contain absorption lines, Fig. S1b. (This procedure is possible in rare-earth doped crystals characterized by narrow absorption lines and spectral ranges with practically no absorption, $k_{abs}l$<<1, between the groups of lines.) This scaled transmission τ^c can be viewed as the transmission measured between the two planes situated just inside the front and the rear walls of the sample. The common (although not precisely correct) practice is to retrieve the spectrum of absorption coefficients $k_{abs}(\lambda)$ using the formula

$$k_{abs}(\lambda) = -\ln(\tau^c(\lambda)/l), \qquad \text{(S13)}$$

where l is the length of the crystal. As one can see in Fig. S1a, the retrieved *approximate* spectrum $k_{abs}(\lambda)$ is almost indistinguishable from the original *exact* spectrum $k^e_{abs}(\lambda)$. The retrieved spectrum $k_{abs}(\lambda)$ can be substituted to Eqs. 3, 4 and 1c, to calculate the *approximate* spectra $\rho(\lambda)$, $\tau(\lambda)$ and $\alpha(\lambda)$, which are also almost the same as the original *exact* spectra $\rho^e(\lambda)$, $\tau^e(\lambda)$ and $\alpha^e(\lambda)$, Fig. S1b. This good agreement proves high accuracy of the approximate retrieval method described above. We use this technique in our studies to retrieve the spectra $k_{abs}(\lambda)$ and $\alpha(\lambda)$ from the experimentally measured normal incidence transmittance spectra of the Er doped crystals (see Sections 2.2 and 6.3).

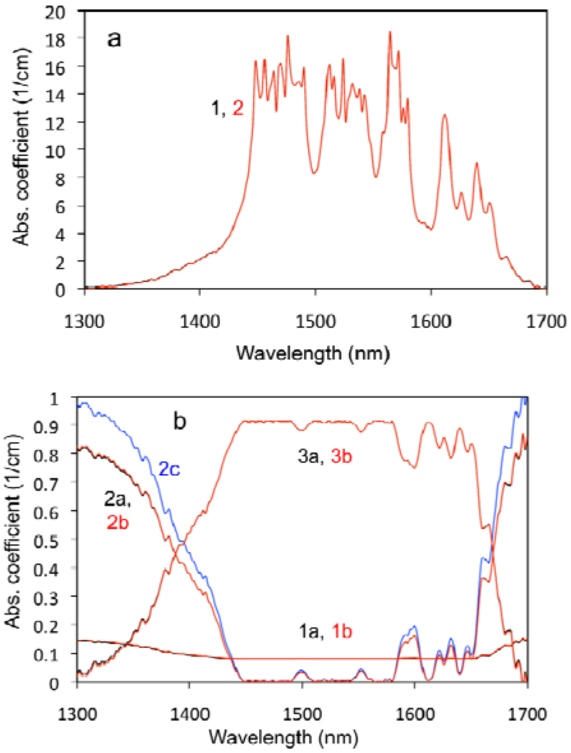

Fig. S1. (a) The original spectrum of absorption coefficients $k^e_{abs}(\lambda)$ (trace 1) and that retrieved using the approximate method (Eq. S13). The two spectra are almost on the top of each other. (b) The exact spectra $\rho^e(\lambda)$, $\tau^e(\lambda)$ and $\alpha^e(\lambda)$ (traces 1a, 2a and 3a, respectively) calculated using the spectrum of absorption coefficients $k^e_{abs}(\lambda)$. The analogous spectra $\rho(\lambda)$, $\tau(\lambda)$ and $\alpha(\lambda)$ (traces 1b, 2b and 3b, respectively) calculated using the spectrum of absorption coefficients $k_{abs}(\lambda)$. The scaled transmittance spectrum $\tau^c(\lambda)$, calculated as described above Eq. S13 (trace 2c).

References

(60) Siegman, A. E. *Lasers* (Kelly, A.) p. 1283 (University Science Books, 1986).

(61) Koechner, W. & Bass, M. *Solid State Lasers: a graduate text (eds.* Koechner, W. and Bass, M.*)* p. 409. (Springer, New York, 2003).

(62) Sivukhin, D. V. *General course of physics, Vol. 4, Optics* (in Russian).

(63) Pantell, R. H. & Puthoff, H. E. *Fundamentals of Quantum Electronics* (John Wiley & Sons Inc., 1969).
